# Study design to develop and pilot-test a web intervention for partners of military service members with alcohol misuse

**DOI:** 10.1186/1940-0640-9-18

**Published:** 2014-09-02

**Authors:** Karen Chan Osilla, Eric R Pedersen, Kristie Gore, Thomas Trail, Stefanie Stern Howard

**Affiliations:** 1 RAND Corporation, 1776 Main Street, P.O. Box 2138, Santa Monica, CA 90407-2138, USA; 2 RAND Corporation, 1200 South Hayes Street, Arlington, VA 22202-5050, USA

**Keywords:** Military spouse, Alcohol misuse, Unhealthy alcohol use, Web intervention, Computer-assisted intervention, CRAFT, Spouse or significant other

## Abstract

**Background:**

Alcohol misuse among military service members from the recent conflicts in Iraq and Afghanistan is over two times higher compared to misuse in the civilian population. Unfortunately, in addition to experiencing personal consequences from alcohol misuse, partners and family members of alcohol-misusing service members also suffer in negative ways from their loved one’s drinking. These family members represent important catalysts for helping their loved ones identify problem drinking and overcoming the barriers to seeking care. This paper describes the protocol to a pilot study evaluating a 4-session, web-based intervention (WBI) for concerned partners (CPs) of service members with alcohol misuse.

**Methods/design:**

The WBI will be adapted from the Community Reinforcement and Family Training (CRAFT) intervention. In the first phase, we will develop and beta-test the WBI with 15–20 CPs. In the second phase, we will randomize CPs to WBI (n = 50) or to delayed-WBI (n = 50) and evaluate the impact of the WBI on CPs’ perceptions of service member help-seeking and drinking, as well as the CP’s well-being and relationship satisfaction 3 months after the intervention. In the third phase, we will recruit 15–20 service members whose partners have completed the study. We will interview the service members to learn how the CP-focused WBI affected them and to assess whether they would be receptive to a follow-on WBI module to help them.

**Discussion:**

This project has the potential to benefit a large population of military service members who may be disproportionately affected by recent conflicts and whose drinking misuse would otherwise go undetected and untreated. It also develops a new prevention model that does not rely on service members or partners attending a hospital or clinical facility to access care.

**Trial registration:**

NCT02073825.

## Background

U.S. military service members with alcohol misuse are a vulnerable population with a high unmet need for intervention efforts [1]. Alcohol misuse occurs before more severe sub-classifications of an alcohol use disorder and can be quantified as at-risk or heavy drinking (more than 3 drinks/day or 7 drinks/week for women; more than 4 drinks/day or 14 drinks/week for men) [2,3]. Approximately 40 percent of the nearly 775,000 married service members in the U.S. Armed Forces report heavy drinking [4]—a rate over twice that of the 13 percent of married civilians reporting heavy drinking [5]. For the service member, alcohol misuse is associated with numerous occupational, relational, and personal consequences (e.g., fitness for duty, absenteeism, comorbid depression and anxiety [1,6,7]). For the family of these service members, alcohol misuse is correlated with poor marital quality, greater rates of infidelity and separation/divorce, intimate partner violence, and child maltreatment [8,9], and alcohol is involved in approximately one-quarter of emotional and physical abuse incidents in this population [10].

Current military policies that mandate reporting to commanding officers may prevent service members from seeking specialty care services for alcohol use disorders. Service members and their partners are often reluctant to seek out services due to the lack of confidentiality and negative repercussions associated with seeking care for alcohol misuse in the military (e.g., appearing on their record [11,12]). Indeed, guardsmen report that their greatest concern with seeking care for alcohol misuse is fear it would appear on their record [11]. Military reporting policies [1,13], which may require health care providers to report alcohol use disorder diagnoses to an individual’s commanding officer, may not only prohibit treatment-seeking, but may also escalate problematic drinking patterns and potentially impact operational readiness for the service member and their unit [1]. These prohibitive barriers have made the prevention and early intervention of alcohol misuse among service members a difficult challenge to overcome.

Although alcohol misuse can weigh heavily on service members’ relationships, spouses or partners of service members can also be an important catalyst for motivating service members to seek treatment. Spouses/partners are in close proximity to and spend significant time with the service member; they are also motivated and typically want to help their partner reduce drinking to improve their relationship as well as to alleviate their own struggles resulting from their partner’s drinking [14,15]. The concerned partner (CP) may be more likely to recognize warning signs of misuse, compared to the service members, who may be more resistant to admitting at-risk drinking issues [16,17]. Service members with alcohol misuse report encouragement from partners as the most prevalent facilitator of pursuing care [11], and individuals changing drinking patterns most often cite partner support as the most helpful mechanism in supporting change [18]. Accessible prevention-focused programs for the CP can encourage help-seeking among service members, alleviate mental health symptoms of partners, and increase relationship satisfaction [19-21]. CPs can encourage their partners to seek help to prevent the progression to dependence. CPs also can seek help for themselves to develop boundaries, coping, positive communication skills, and an awareness of how their behavior may be reinforcing the drinker’s continued problematic use (e.g., calling in sick for them after a night of heavy drinking [22-24]). If the relationship is strained by the alcohol misuse, a CP-focused intervention could present an opportunity to improve the quality of the relationship and the perceived support both the CP and the service member experience.

Many existing couples-based interventions in nonmilitary cohorts focus on partners with already-established alcohol use disorders, but they are labor-intensive and are delivered face to face [25-27]. Other less intensive CP interventions have traditionally been based on the 12-step approach (e.g., Al-Anon) or the Johnson intervention (i.e., confronting the family members during a structured, often surprise, group session; [28,29]). Al-Anon is a 12-step worldwide support group for relatives and friends of individuals with alcohol problems. These intervention approaches focus on the CP’s well-being and “loving detachment” from their loved one’s drinking [30]. Another approach, the Community Reinforcement and Family Training (CRAFT) intervention [24], supports CPs in making positive changes in their own lives (e.g., engaging in pleasurable activities, seeking support), while teaching them to effectively communicate with their partner so the partner might consider seeking treatment. CPs engaged with the CRAFT model first learn how to change their own lifestyle and take care of their own needs. They receive feedback about enabling behaviors that may unintentionally reinforce continued abuse of alcohol by their partner; they establish new communication skills to interact with their loved ones to help them consider change; and they learn how to change the environment of their partner to create a nonsubstance using lifestyle that is more rewarding than one focused on using alcohol [20,24,31].

While CRAFT, the Johnson intervention, and Al-Anon all have been associated with significant improvements in depression, anger, relationship satisfaction, and family conflict among CPs, CRAFT consistently has been shown to increase engagement in alcohol-focused treatment two to three times more than these other comparison interventions [19-21]. While efficacious, CRAFT is typically conducted face to face, which somewhat limits the number and extent of individuals that can be reached using the approach. Specifically, for military CPs, a face-to-face intervention may not be an option due to such barriers as confidentiality, concerns within the military behavioral health system, and frequent moves due to deployment. Thus, more accessible programs based on empirically supported interventions such as CRAFT are needed to reach this at-risk population in their homes—confidentially and on their own time—to help avoid more serious alcohol problems from developing.

Web-based interventions (WBIs) are emerging practical mechanisms for reaching individuals struggling with substance use who may experience barriers to care. WBIs have been increasingly used for alcohol misuse because of decreased stigma compared to formal treatment programs; they also can safeguard anonymity and privacy and are geared toward self-guided pacing [32,33]. WBIs can extend the reach and impact of existing preventive interventions. That is, they can assist those at the beginning stages of alcohol misuse to help prevent development of long-term problems or dependence. Although there have been several alcohol-related WBIs developed for alcohol misuse among younger populations, all have been individually focused on the person with misuse; most involve a nonmilitary college population, and none are directed to the CP [34-36]. Additionally, most existing web-based approaches that target couples are conjoint and focus on general relationship satisfaction, not alcohol misuse [37]. Currently, no WBI model exists to help CPs prevent alcohol misuse among their service member family members, so this study represents an important service need of the military community.

There is preliminary evidence that web-based approaches for use by CPs would be well-accepted and accessible to the underserved population of CPs and substance-using partners. Specifically, Rychtarik and colleagues [38] assessed interest in and accessibility to a coping skills program designed for female CPs of people with problem drinking. The majority of women (66%) had never sought help for their partner’s alcohol misuse (nor had their partners) and over three-quarters (77%) reported either preferring an online intervention of this type or rated it equally preferential to face-to-face formats (77%). It is possible that a web program tailored specifically for CPs of service members may be acceptable and even preferred over a face-to-face intervention.

In the current randomized controlled pilot study, *Partners Connect*, we will adapt CRAFT as a WBI for CPs living with service members who have alcohol misuse problems and evaluate the WBI compared to delayed-WBI at 3-month follow-up. Based on feedback from this study, we will develop a follow-on WBI module for use by the service members themselves. We will utilize the evidence-based CRAFT approach to serve as a model for intervention content, and the WBI will consist of four modules designed to be completed at the CP’s own pace. This study represents the first WBI for concerned military partners that targets service member alcohol misuse. It is self-sustainable and flexible, and thus expected to extend the reach and impact of existing preventive interventions. This study will fill an important gap in the alcohol misuse prevention literature and also help establish evidence that service members are willing to seek care and reduce drinking when their CPs are given resources to help them.

## Methods/design

### Overview of *Partners Connect*

The *Partners Connect* study will develop and pilot-test a CRAFT-adapted WBI for CPs of active duty service members (Table 1). In Phase 1, our formative assessment, we will recruit 15–20 CPs from Facebook Family Readiness Group (FRG) pages and conduct iterative beta-testing of the WBI program with CPs. FRGs are geographically specific groups composed of service members’ family members. Group leaders have formed Facebook pages for their local FRGs to make announcements, promote events, and provide support for each other. In Phase 2, we will pilot-test the final WBI developed in Phase 1. We will randomly assign CPs to WBI or delayed-WBI and assess outcomes 3 months after the intervention. We hypothesize that, compared to CPs in the delayed-WBI (n = 50), WBI CPs (n = 50) will report increased relationship satisfaction and improved health, and report perceived reductions in their partner’s drinking at the 3-month follow-up. In Phase 3, we will interview service members (n = 15–20) to better understand how the CP-focused WBI affected them and assess whether they would be receptive to a follow-on service-member WBI module. We will ask service members their intervention preferences and obtain feedback on a planned WBI. These data will inform the development of a new module for service members that we hope to evaluate in a future trial.

**Table 1:** Study phases and milestones

**Phase**	**Milestones**
1	• Develop a 4-session WBI prototype
	• Beta-test WBI prototype and survey/interview 15–20 CPs
	• Finalize WBI for randomized controlled trial
2	• Randomize 100 CPs to WBI or delayed-WBI
	• Assess 100 CPs at 3-month follow-up
3	• Recruit 15–20 service members of WBI CPs that have completed follow-up
	• Conduct phone interviews with 10–15 service member regarding a follow-on service member module

Note: WBI = web-based intervention; CP = concerned partner.

### Participants

#### Concerned partners

Individuals will be eligible for this study if they meet the following eligibility criteria: 1) currently living with their partner; 2) have had contact with their partner at least 40 percent of the time in the past 90 days (e.g., most of the evenings or most of the days in a given week); 3) in a romantic relationship with the service member; 4) have a computer with internet access they can use in a private area; 5) the CP and service member are at least 18 years of age; 6) their partner is currently an active duty service member; 7) the CP or service member has not attended couples or drug/alcohol counseling in the past 60 days; 8) the CP does not plan to separate from their partner in the next 60 days; 9) the CP thinks their partner has a drinking problem; and 10) the CP does not report any domestic violence in the past year. The purpose of these eligibility criteria is to recruit CPs who are not in treatment, who are in frequent contact with their partner (to increase reliability of collateral report), and who feel safe participating in the study. These criteria have been adapted from previous CRAFT clinical trials [19-21].

#### Service members

Service members will qualify for Phase 3 of the study if their CP completed all four WBI usability surveys (an indicator of completing all WBI sessions). We anticipate service members to be male and an average age of about 30 years [39]. No additional inclusion and exclusion criteria will be applied because we are interested primarily in service members of CPs who have completed the WBI.

### Recruitment

Participants in Phases 1 and 2 will be recruited through Facebook FRG pages in two ways. There currently are over 100 region-specific FRGs on Facebook, with between 30 and 500 members each. We will recruit CPs by: 1) asking FRG administrators for their permission to post study information on their Facebook page; and 2) posting advertisements for FRG Facebook members.

Facebook FRGs are private groups hosted on Facebook and maintained by family members themselves. Each official Facebook FRG page has an administrator (i.e., family member of a service member) that maintains the site, accepts other FRG members on Facebook into the group, posts announcements for events, and facilitates connections between other FRG members on the site. We will ask the administrators of these Facebook group pages to let group members know about an opportunity to participate in our study.

Facebook recruitment strategies such as these have successfully recruited large samples of military and civilian samples for research studies [40-43]. This type of recruitment method has also been successful in prior research with young adults and has the ability to recruit nationally representative samples for generally low cost [40,43]. Based on previous studies, we conservatively anticipate being able to recruit 100 participants in less than 3 months. For example, Brief et al. [41] recruited 600 participants in 6 weeks using Facebook recruitment methods only. Female CPs are estimated to make up the entire sample based on other studies recruited from FRGs [44]. CPs are estimated to be an average age of 27 and primarily Caucasian (66%), with smaller proportions of Hispanic (18%), African American (11%), Asian/Pacific Islander (3%), Native American (1%), and Other (1%) [44].

**Figure 1:**
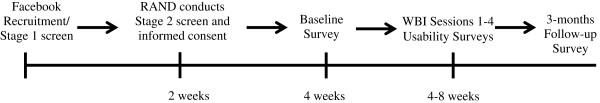
Intervention and data collection flow.

Interested individuals will be asked to participate in a two-stage screening (Figure 1). The first stage will be online, which will consist of the first six eligibility criteria stated above. If eligible based on the stage 1 screen, the CP will be asked to complete a consent-to-contact form that includes their contact information to learn more about the study. In a second-stage screen, research staff will phone the CP to describe the study, conduct additional eligibility screening (the last three eligibility criteria stated above), and administer informed consent. We will offer a list of resources (e.g., Military OneSource; Substance Abuse and Mental Health Services Administration’s Treatment Referral Line) to any CPs who are not eligible to participate.

In Phase 3 of the study, service members will be recruited via CPs in Phase 2. CPs will be asked during their follow-up survey if it would be acceptable to contact their service member partner for a brief, 30-minute individual phone interview. Study staff will subsequently contact service members, describe the study, and ask for their verbal consent to an audiotaped interview by phone.

### Description of the WBI

The main goals of the WBI are to: 1) help the CP increase their own happiness in various aspects of their lives; 2) teach the CP how to manage their own behavior (e.g., communication) toward their service member partner; and 3) identify ways the CP can help the service member reduce their drinking [24]. The WBI is under development, but will include four sessions that will take about 30–45 minutes each depending on the CP’s pace. Phase 1 of the study will be focused on beta-testing the WBI with CPs. While the delivery and messaging of the WBI will likely change as we beta-test it, the main content is empirically based on CRAFT [24,31] and will not change.

#### WBI content

The first session of the WBI will focus on CP self-care by identifying areas in their life they want to improve upon and, strategies/activities to help improve these areas. CPs will also be encouraged to identify a support person they trust, someone they can tell about the program and also with whom to practice some of the skills they learn. The second session will focus on helping the CP with positive communication skills. The CP will view video vignettes of characters identifying old communication patterns (i.e., “broken records”) that usually come up in their relationship (e.g., partner comes home late at night, CP is upset because she had dinner ready and yells at him, partner yells back) and using positive communication to develop new patterns. The third session will focus on conducting a modified functional analysis of the service member’s drinking, an integral part of the CRAFT approach, to help the CP identify reasons why the service member may be drinking (e.g., to manage negative emotions like anxiety or depression) and to identify positive, alternative, nondrinking reinforcers in the service member’s life. Finally, the fourth session will provide further practice of communication strategies, discuss how to withdraw positive reinforcement when their partner drinks, and prepare the CP to transition to next steps (e.g., if the CP wants to suggest help-seeking to partner or wants to help partner reduce drinking). Each session ends with a session summary and a list of practice activities, which are reviewed for completion at the beginning of subsequent sessions.

#### WBI delivery

The WBI content will be delivered using Motivational Interviewing (MI; [45]) and Cognitive Behavioral Therapy (CBT; [46]) principles. Each session will have a mix of video and audio components. A narrator will lead the CP through each session by discussing session goals, providing feedback, summarizing the session and practice assignments, asking the CP open-ended questions, and directing the CP to click on videos and other elements of the WBI. To integrate MI into our WBI, we will include open-ended questions, affirmations, reflective statements, and summaries so that the intervention feels collaborative and supportive of the CP [47]. For example, we will present lists of options for the CP to choose from (e.g., Here are a list of strategies that have helped other CPs; which would you be willing to try before your next session?). Where possible, we will use importance and willingness rulers to elicit change talk (e.g., Why a 4 and not a 0? Type your response below.). The WBI will also utilize digital storytelling [48,49], where CPs can click on videotaped vignettes of military partners who may be going through similar issues. This method is currently being utilized by the military [50] to address stigma and increase treatment utilization among veterans. These videotaped vignettes will demonstrate teaching points (e.g., positive communication) and provide examples of CPs going through similar experiences (e.g., a CP who has little social support due to numerous relocations and deployments).

The WBI is designed to be self-contained, self-administered, and accessed from any Internet-connected computer. The WBI requires the CP to enter a unique password to access the program. All data transmitted through the surveys and WBI will be encrypted over the Internet, not saved locally onto the CP’s computer, and then housed in a HIPAA-compliant MySQL database that is protected by a 128-bit encrypted password. These safeguards ensure confidential data transmission and storage.

### Procedures

#### Phase 1

The purpose of Phase 1 is to assess the feasibility of our recruitment and data collection protocol; adapt the CRAFT for CPs of service members (n = 15–20); and receive feedback from them to determine if it is appropriate and helpful. We will implement usability testing procedures assessing ease of use, acceptability (whether the session is agreeable or satisfactory), appropriateness (whether the WBI is relevant and compatible with their issues), and feasibility (whether the WBI can be successfully used and completed) from our previous WBI work with various populations [47,51]. The beta-testing phase will occur in three steps. In the first step (after stage 2 eligibility screening), research staff will direct the CP to create a password to access the consent form and baseline survey and to authenticate the user. In the second step, after the CP completes the consent and baseline survey, the CP will be automatically directed to the first WBI session or offered an option to schedule an alternate time for completing it. CPs will be asked to complete all four sessions, ideally spaced 10 days to 2 weeks apart. After each session, there will be a short usability survey assessing the acceptability and usability of the session (e.g., On a 1–10 scale, how helpful/unhelpful was the session? What was difficult to understand? What might you change?). In the third step, after the CP has completed all four WBI sessions, a study staff member will conduct a 30-minute telephone interview with the CP to obtain the CPs’ general impressions of the program, ask if the CP practiced any of the session material with the partner and what impact that had, and elicit suggestions for what might improve the program and be more helpful for other CPs. We will also assess acceptability (How much did you agree with the information shared with you?), appropriateness (How relevant was the information to your relationship and your partner’s drinking?), and feasibility (How was it completing the WBI on your own computer? How was the length of each session and the number of the sessions? What made it difficult/easy to complete?).

Phone conversations will be audiotaped, transcribed, and reviewed to identify common themes, and the information collected will be used to revise the WBI prototype. We will therefore be able to revise the WBI on an ongoing basis. For example, we will ask questions about words that are difficult to understand. Answers to these questions will allow us to implement any suggested changes before we proceed with the next CP so that revisions can also be beta-tested.

##### Phase 1 analysis plan

Following grounded theory analyses [52], key points with similar themes will be grouped together into a category if mentioned several times by the CPs (e.g., Learning to communicate my concerns was helpful) [53]. Two staff members will then discuss each of the categories and generate underlying themes from the data (e.g., Sharing my worries with my partner drew us closer). After themes are extracted, content analysis will be used to identify quotations that fit each of the themes [54,55]. Then, we will independently sort quotations by theme and reach a consensus on any discrepancies. This analysis will be performed to understand both the feasibility and acceptability of the WBI for a CP population and to inform the delivery of the WBI in Phase 2.

#### Phase 2

Participants (N = 100) will be recruited as in Phase 1 from multiple Facebook FRG group pages. CPs will be randomly assigned to WBI or to the delayed-WBI after completion of their baseline survey, using computerized permuted block randomization with random size blocks, thereby ensuring the number of people allocated to each group is approximately equal throughout recruitment [56].

Table 2 displays our assessment measures. Data will be collected from CPs at baseline, after each WBI session, and 3 months after the fourth WBI session (or 4 months post-baseline for those who do not complete the WBI or who are in the delayed-WBI condition). Research staff will also schedule the WBI sessions with CPs during the consent process, asking them to complete one session every 10 days. Staff will monitor all participants through an online dashboard that tracks when surveys (and WBI sessions) are accessed and completed. If a CP has not completed a survey or session, staff will send e-mail reminders to complete the session and will follow up with phone calls as needed.

**Table 2:** Baseline and 3-month follow-up assessment measures completed by CP

**Measure**	**Baseline**	**3 months**
Demographic information^1^	X	
CP concern about service member’s drinking^2^	X	X
Perceptions of service member alcohol use^2^	X	X
Service member’s perceived readiness to change drinking behavior and/or seek help^2^	X	X
Relationship quality^2^	X	X
Amount and quality of communication with service member about drinking^2^	X	X
CP’s behaviors around service member’s drinking (e.g., encouraging, enabling)^3^	X	X
Family conflict and cohesiveness^2^	X	X
CP alcohol use^3^	X	X
CP depressive symptoms^3^	X	X
CP anxiety symtpoms^3^	X	X
CP mental health service use^3^	X	X
CP general health and well-being^3^	X	X
WBI session usability measures^3^	X	X
Relationship communication style^1^	X	X

^1^ = covariate, ^2^ = primary outcome, ^3^ = secondary outcome.

Partners randomized to the delayed-WBI condition will be invited to complete the same WBI program after they complete their 3-month follow-up. This allows for all CPs to receive the WBI information after their study assessments have been completed. We will not assess outcomes in the delayed-WBI because this extends beyond the scope and budget of this pilot study. While the WBI is unproven for this population, the concepts are evidence-based from CRAFT and have been shown to be helpful to CPs [19-21]. Because participants report concern about their loved ones’ drinking, we feel it is important to provide them with the intervention information should they elect to receive it.

##### Phase 2 analysis plan

Analyses will use the standard intent-to-treat (ITT) approach to examine the effect of offering the WBI to all CPs. Our ITT approach will analyze CPs as belonging to the group they were randomized to, regardless of their compliance, because excluding CPs that do not complete their WBI sessions would bias our results in favor of the WBI and increase the type I, or false positive, error rate [57].

Our proposed primary outcomes will be to examine if CPs report fewer concerns about their partner’s drinking [58] and measure perceived reductions in their partner’s drinking [59], perceived increases in their partner’s readiness to change or seek help, and perceived improvement in relationship and communication quality [58,60,61]. These measures will be beta-tested in Phase 1 (see Table 2).

The data will be analyzed with an aim of providing information to improve the planning of a larger trial. We will examine the distributions of variables and consider the implications for refining these measures (e.g., to eliminate floor and ceiling effects). Our primary analysis will be to examine differences in outcomes between WBI and the delayed-WBI group 3 months after the intervention. For continuous outcomes, we will use the SAS PROC MIXED procedure to analyze changes in outcome variables from baseline to follow-up. PROC MIXED is a robust procedure used to determine missing values and correlations between outcome variables, so it has been recommended for the analysis of ITT designs [62]. For categorical outcomes, we will use the SAS PROC GLIMMIX procedure, which estimates mixed models with binomial outcomes. If the sample is demographically diverse (by age, race/ethnicity), then we will control for these characteristics.

We will also carry out an exploratory analysis to see if changes in alcohol use are accompanied by changes in CP reports of relationship satisfaction. Previous research has established that alcohol misuse is correlated with relationship dissatisfaction [63], but this analysis will demonstrate whether a decrease in alcohol use by the service member is associated with increases in relationship satisfaction compared to baseline. We will also explore relationship satisfaction, CP alcohol consumption, and couples communication style as moderators of the effectiveness of the CP’s WBI. Separate models will be utilized to assess changes in perceptions of service members’ alcohol use as the product of the intervention condition and each moderating variable. Specifically, we will enter the interaction of each of these variables with intervention condition into separate mixed models for predicting pre/post changes in perceived service member alcohol use. Such analyses are underpowered for conventional hypothesis tests and would therefore be preliminary.

#### Phase 3

The goals of Phase 3 are twofold: 1) to gather feedback on how service members responded to their partners completing our WBI; and 2) to assess whether service members would be receptive to completing a WBI module or other intervention tailored to their drinking. Including a service member component in this study is essential to understanding the impact of the CP-focused WBI. This information can also elucidate CP outcomes (e.g., if we find that CPs reported improved communication with their partner regarding drinking, we can corroborate this information with service members). In addition, if we find that service members reacted negatively to their CP receiving the WBI, this would be helpful information to inform future iterations of the CP-focused WBI. Finally, anything service members found helpful from their CP attending the WBI may also be used to inform future development of a service member-focused WBI. For example, we will provide examples of a service member-focused WBI and if the service members provide feedback to us that it is too lengthy or burdensome, we would use this knowledge to help restructure the service member WBI accordingly.

While the construction of the add-on service member intervention module would be out of scope and budget for the current study, the results of this phase would directly prepare us for a future trial that evaluates a larger randomized controlled study of a WBI for CPs with a follow-on intervention for service members.

During phone interviews (~30 minutes) with the service members, we will assess the impact of the WBI on the service member (e.g., What do you know about the web program your partner completed? What differences, if any, did you notice in your partner? What do you think about those differences?). We also will assess how receptive they are to completing a WBI tailored specifically for them (e.g., There are a few options for help-seeking I’d like to discuss with you. If a confidential web program was made available to you, what’s the likelihood you would check it out? What factors would make it more likely that you would check it out [e.g., confidential, not tied to military reporting]? What factors would make it more difficult? What types of information would you want to know about? What types of information would “turn you off?” May I describe a couple of examples to get your feedback on how these can be improved?).

##### Phase 3 data analysis

Our qualitative analysis plan will be similar to that in Phase 1. Interviews will be audiotaped and coded by identifying, labeling, and grouping together key points that speak to the feasibility and acceptability of adding a WBI module for service members. Two research staff members will review, sort, discuss, categorize, and generate underlying themes from the data.

### Alternative designs we considered

We deliberated on a number of research designs to optimize scientific rigor and decided on a combination of qualitative and quantitative methods. We considered a parallel-arm, randomized trial comparing WBI to no WBI, but felt this potentially would take away helpful information for CPs in the comparison group. Thus, we proposed a delayed-WBI condition. We also considered conducting another follow-up with the delayed-WBI condition to assess differences before and after the WBI, but the logistics of this added procedure would be out of scope and budget for this pilot study. We also considered recruitment through primary care at medical treatment facilities or through in-person recruitment of FRGs at military installation bases. This design would have allowed us to recruit in person, but would have localized and narrowed recruitment to one base, would be less efficient and less generalizable than Facebook recruitment, and may have been challenging due to the stigma surrounding alcohol misuse. Finally, we considered a couples-oriented WBI, but anticipated several problems documented in the literature, including practical barriers involved with having two people complete a WBI simultaneously (e.g., different reading speeds, needing to coordinate schedules, a partner being out of town); potential conflict that could result without proper advanced CP training (e.g., a CP saying “I told you so” and the service member becoming more resistant); and less time to individually reflect on the intervention information [37]. Therefore, we feel that our current research design optimizes the proportion of CPs and service members who could benefit from this preventive intervention.

## Discussion

The current study addresses an important public health problem: alcohol misuse among military service members. We will develop a preventive intervention, an early intervention model recommended recently by the Institute of Medicine [1]. The WBI will be unique because it targets the CP, is web-based, and exists outside the traditional health care system as a stand-alone health promotion intervention. Our study will also develop a sustainable protocol for ongoing CP intervention if shown to be efficacious, and it will derive important data on service member intervention preferences to inform the development and testing of a follow-on WBI module for service members.

## Competing interests

The authors declare that they have no competing interests.

## Authors’ contributions

KCO, ERP, TT, and KG conceptualized the study and obtained funding. KCO has overall responsibility for the execution of the WBI intervention, data collection, analyses, and reporting. KCO and ERP conducted literature searches and provided summaries of previous research studies. ERP will assist with the design and evaluation of the WBI. TT will perform quantitative data analyses. KG will assist with the design of Phase 3. SSH contributed to the draft of the manuscript and will assist with study coordination, data collection, and qualitative data analyses. All authors read and approved the final manuscript.
